# The Complete Chloroplast Genome of Banana (*Musa acuminata*, Zingiberales): Insight into Plastid Monocotyledon Evolution

**DOI:** 10.1371/journal.pone.0067350

**Published:** 2013-06-28

**Authors:** Guillaume Martin, Franc-Christophe Baurens, Céline Cardi, Jean-Marc Aury, Angélique D’Hont

**Affiliations:** 1 CIRAD (Centre de coopération Internationale en Recherche Agronomique pour le Développement), UMR AGAP, Montpellier, France; 2 Genoscope, Evry, France; Donald Danforth Plant Science Center, United States of America

## Abstract

**Background:**

Banana (genus *Musa*) is a crop of major economic importance worldwide. It is a monocotyledonous member of the Zingiberales, a sister group of the widely studied Poales. Most cultivated bananas are natural *Musa* inter-(sub-)specific triploid hybrids. A *Musa acuminata* reference nuclear genome sequence was recently produced based on sequencing of genomic DNA enriched in nucleus.

**Methodology/Principal Findings:**

The *Musa acuminata* chloroplast genome was assembled with chloroplast reads extracted from whole-genome-shotgun sequence data. The *Musa* chloroplast genome is a circular molecule of 169,972 bp with a quadripartite structure containing two single copy regions, a Large Single Copy region (LSC, 88,338 bp) and a Small Single Copy region (SSC, 10,768 bp) separated by Inverted Repeat regions (IRs, 35,433 bp). Two forms of the chloroplast genome relative to the orientation of SSC versus LSC were found. The *Musa* chloroplast genome shows an extreme IR expansion at the IR/SSC boundary relative to the most common structures found in angiosperms. This expansion consists of the integration of three additional complete genes (*rps15*, *ndhH* and *ycf1*) and part of the *ndhA* gene. No such expansion has been observed in monocots so far. Simple Sequence Repeats were identified in the *Musa* chloroplast genome and a new set of *Musa* chloroplastic markers was designed.

**Conclusion:**

The complete sequence of *M. acuminata* ssp *malaccensis* chloroplast we reported here is the first one for the Zingiberales order. As such it provides new insight in the evolution of the chloroplast of monocotyledons. In particular, it reinforces that IR/SSC expansion has occurred independently several times within monocotyledons. The discovery of new polymorphic markers within *Musa* chloroplast opens new perspectives to better understand the origin of cultivated triploid bananas.

## Introduction

Chloroplasts are the photosynthetic organelles that provide energy for plants and algae. They are also involved in major functions such as sugar synthesis, starch storage, the production of several amino acids, lipids, vitamins and pigments and also in key sulfur and nitrogen metabolic pathways. In angiosperms, chloroplastic (cp) genomes exist at least in part as a circular DNA molecule [1] ranging from 120 to 160 kb in length. Most cp genomes have a quadripartite organization comprising two copies of 20 to 28 kb Inverted Repeats (IRs) which separate the rest of the genome into a 80–90 kb Large Single Copy region (LSC) and a 16–27 kb Small Single Copy region (SSC) [2]. In angiosperms, the cp genome usually encodes 4 rRNAs, 30 tRNAs, and about 80 unique proteins. Earlier studies, using restriction site mapping, have demonstrated that gene content, gene order, and genome organization are largely conserved within land plants [3], [4]. However, with the increasing number of whole cp genome available, many structural rearrangements, large IR expansion and gene loss have been reported [2], [5], [6]. These events can be used for the reconstruction of plant phylogeny [7]. Besides, the availability of whole chloroplast genomes or complete sets of cp genes have helped resolving relationships among major clades of angiosperms [8], [9] with more accuracy than even well-chosen “Lucky Genes” [10]. Most of the reported complete monocotyledons chloroplast genomes are from the Poales group (so far 31 of the 46 complete chloroplast genomes deposited in Genbank). It is thus important to have more representatives of other clades to better understand the evolution of cp genome within monocots.

Bananas (genus *Musa*, family Musaceae) are monocotyledons from the Zingiberales, a sister group of the Poales. Banana is of major economic importance in many tropical and subtropical countries where it is vital for food security and also a major source of incomes. Bananas are widely exported to industrialized countries where they represent the most popular fruit. A reference sequence of *Musa acuminata* nuclear genome has recently been published based on sequencing a DNA extract enriched in nucleus [11], yet providing additional sequence data to assemble a chloroplastic genome. In banana, the peculiar paternal inheritance of the mitochondrial genome associated to the classic maternal inheritance of the chloroplast genome [12] make cytoplasmic markers potentially very useful for analyzing the origin of cultivars, most of which are spontaneous triploid inter-(sub)-specifc hybrids [13]–[15]. In previous studies based on RFLP [16] or PCR-RFLP [17] a total of nine different chloroplastic patterns have been identified among cultivated bananas and related wild species. However, most *M. acuminata* sub-species and cultivars had identical pattern restraining the identification of cultivars progenitors [18].

In this study, we report the assembly, annotation and structure analysis of the complete cp genome of banana. We compare its organization (gene content, IR expansion/contraction, structural rearrangement) with the complete genome of 34 monocots and 10 more basal angiosperms. We also provided new cp markers designed from Simple Sequence Repeats (SSR).

## Materials and Methods

### Sequence Data

A reference nuclear genome sequence of the doubled-haploid Pahang accession (DH-Pahang) was produced based on DNA extraction enriched in nuclear content. A total of 27,495,411 reads were generated using Roche/454 GSFLX pyrosequencing platform. An addition of 1,069,954 paired-Sanger 10 kb insert-size reads and 49,216 paired-Sanger BAC-ends sequenced on two BAC libraries generated with *Hind*III and *Bam*HI restriction enzymes were produced [11].

The plastid reads were extracted from the total using blast similarity search against *Phoenix dactylifera* whole chloroplast genome (NC_013991). The 454 filtered reads were then assembled into sequence contigs using *de novo* assembly with Newbler. A total of six contigs were obtained. Using a python script, an iterative elongation for both ends of each contig using the total 454 reads was applied to ensure that contribution of *Musa* specific sequences was taken into account. The resulting four contigs (one contig for each region except two for the IRs) were then ordered based on *P. dactylifera* chloroplast structure. A mapping step using the paired-Sanger 10 kb insert-size reads was then applied to confirm and correct contig junctions. A total of 1,800,008 GS FLX Titanium reads and 33,583 paired-Sanger reads were mapped to the assembled plastid genome representing 6.5% and 3.1% of the total 454 and Sanger reads for an average coverage of 5,341 X (sd = 2,048), the large standard deviation mainly due to the doubling of coverage in the IRs. The minimum coverage was 619 X and the maximum coverage was reached in the IRs with a value of 9,500 X. The junction between the two contigs corresponding to the IRs was confirmed with the Sanger reads. The four junctions between the single-copy regions and IRs were confirmed by PCR.

### LSC Orientation Relative to SSC

In order to verify the orientation of the SSC region relative to the LSC region, paired-Sanger BAC-end reads were mapped on the assembled *Musa* chloroplast genome using BLAST. Only pairs presenting more than 90% identity on more than 60% of their length were conserved. An additional filter was applied to conserve only pairs having a mate on the SSC region while the other was on the LSC. A total of 180 paired-Sanger BAC-ends were retained. Orientation visualization of the different paired BAC-ends reads was performed using CIRCOS [19] and was used to infer LSC orientation relative to SSC.

### Genome Annotation

The genome was annotated by using DOGMA [20], followed with manual corrections for start codons. Intron positions were determined based on those of *P. dactylifera* [21] and *Elaeis guineensis* [22]. The transfer RNA genes were annotated using DOGMA and tRNAscan-SE (version1.23) [23]. Some intron-containing genes in which exons are too short to be detected were identified based on comparisons to corresponding exons in *P. dactylifera* and *E. guineensis*. The resulting annotated sequence has been deposited at the European Nucleotide Archive under accession number HF677508.

### Codon Usage

Codon usage frequencies and the relative synonymous codon usage (RSCU) was calculated from coding sequences (CDS) of all different protein coding genes in the *M. acuminata* chloroplast genome using seqinr R-cran package [24].

### Cp DNA Transfers to the Nucleus

Chloroplast DNA transfers to the nucleus were detected using Blast based approach. The assembled *M. acuminata* chloroplast genome, with one of its IR removed, was compared to the 11 chromosomes of *Musa* nuclear reference genome with high stringency blast parameter (e-value <10^−5^, hit length >100 bp). A *per base* insertion value of each plastid base has been calculated as described in The Tomato Genome Consortium [25].

### Phylogenetic Analysis

The phylogeny was performed using 79 plastid protein-coding genes derived from 48 plant species (Table S1) with complete chloroplast sequence, most belonging to monocotyledons. A codon based alignment was performed for each gene using homemade scripts that grouped together homologous genes and then converted them into proteins. An alignment was then applied to the protein sequence using MAFFT [26] and this protein alignment was then used to make the codon based aligment. Each aligned gene was then concatenated into a single matrix. Missing genes were replaced by Ns. A nucleotide matrix of 76,524 sites was then constituted. Evolutionary model choice was performed using jModelTest 2.0.2 software [27]. A maximum likelihood (ML) phylogenetic analysis was then performed using GTR+G+I model of sequence evolution using PhyML v3.0 [28]. Branch support was estimated based on aLRT statistics.

### *Musa* Chloroplast Structure Comparison with others Whole cp Genomes

Gene positions of the different cp genomes were collected from the Genbank file and ordered based on their positions within the genome. Gene order and composition were then compared between the different species. Large events, *e.g.* gene loss, IR gene gain/loss, large structural rearrangement, relative to the basal angiosperm *Amborella trichopoda* [29] were recorded and used to infer scenarios in the different monocot lineages.

### Short Tandem Repeats

Microsatellites (mono-, di-, tri-, tetra-, penta-, and hexanucleotide repeats) detection was performed using MISA [30] with minimum number of repeats of 10, 5, 4, 3, 3, 3 for 1, 2, 3, 4, 5, 6 unit size respectively (Table S2). Minisatellites (unit size ≥10) were detected manually using dot plot with Gepard software [31] with the *Musa* chloroplast sequence plotted against itself. Sequences with unit repeat equal to or higher than 10 bp repeated tandemly at least twice were conserved. The dot plot was inspected on overlapping windows of 5 kbp with an overlap of 1 kbp. Mini- and microsatellites located in the IR regions were only counted once. Primer design was performed using Primer3 software [32] (Table S3).

**Figure 1:**
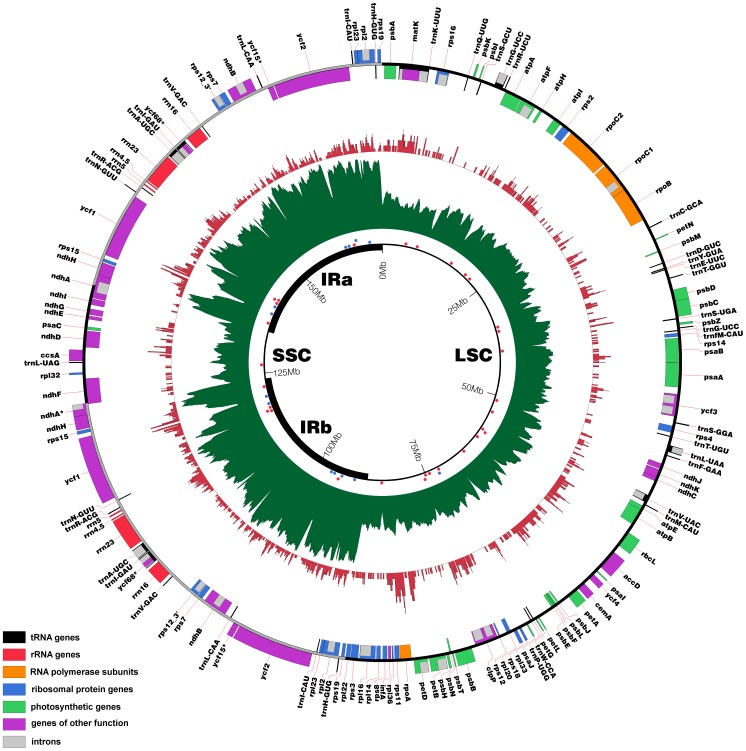
Circular *Musa acuminata* chloroplast map. Genes are represented with boxes inside or outside the circle to indicate clockwise or counterclockwise transcription direction respectively. The color of the gene boxes indicates the functional group to which the gene belongs. Read depth of the genome is represented in the inner green circle. The locations of the short tandem repeats, tested for their polymorphism, are represented with red and blue dots for microsatellites and minisatellites respectively. The *per base* insertion value in the nucleus is drawn in the red circle. The *per base* insertion value of the IR analyzed has been divided by two and applied to both IR. Pseudogenes are marked with asterisks.

A total of 32 SSR located all over the plastid genome (Figure 1) were tested for their polymorphism in a testing panel comprising *M. boman*, *M. balbisiana* and three *M. acuminata* spp., *M.a.* ssp *banksii*, *M.a.* ssp *zebrina* and *M.a.* ssp *malaccensis* (DH-Pahang) with the Applied Biosystems® 3500×L Genetic Analyzer. This set included 7 minisatellites and 26 microsatellites. The 12 most polymorphic markers were evaluated onto 5 additional cultivated accessions, including the triploid accession Cavendish Grande Naine, all belonging to the *Musa* chloroplastic group II [16].

## Results and Discussion

### General Feature of *Musa* Acuminata cp Genome

The *M. acuminata* chloroplast genome is a DNA molecule of 169,972 bp in length. Similar to most other angiosperms, the chloroplast genome of *M. acuminata* is circular with a quadripartite structure: a pair of Inverted Repeats (IRs) (35,433 bp) separated by the Single Copy region (SSC) (10,768 bp) and Large Single Copy region (LSC) (88,338 bp) (Figure 1). A total of 136 functional genes were predicted, including 113 distinct genes comprising 79 protein-coding genes, 30 transfer RNA (tRNA) genes and 4 ribosomal RNA (rRNA) genes (Table 1). All 4 rRNA genes, 8 tRNA and 10 protein-coding genes are repeated in the IR. Protein-coding genes, tRNA and rRNA represent respectively 52.0%, 1.7% and 5.3% of the plastid genome. Non-coding DNA, including intergenic spacers (IGSs) and introns represent 41.0% of the genome. Similar to other plastid genomes, the overall GC content of the *M. acuminata* plastid genome is 36.8%. This value is slightly higher in protein coding genes (37.3%) and introns (37.8%), slightly lower in IGS (31.6%) while tRNA and rRNA show higher GC value with 53.1% and 55.2% respectively.

**Table 1:** *Musa acuminata* plastome characteristics.

Plastome characteristics	
Size (bp)	169,972
LSC size in bp (%)	88,338 (52.0)
SSC size in bp (%)	10,768 (6.3)
IR length in bp	35,433
Size in bp (%) coding regions	100,277 (59.0)
Size in bp (%) of protein-encoding regions	88,336 (52.0)
Size in bp (%) of introns	19,312 (11.4)
Size in bp (%) of rRNA	9,056 (5.3)
Size in bp (%) of tRNA	2,885 (1.7)
Size in bp (%) of IGS	50,389 (29.6)
Number of different genes	113
Number of different protein-encoding genes	79
Number of different tRNA genes	30
Number of different rRNA genes	4
Number of different genes duplicated by IR	24
Number of different genes with introns	18
Overall % GC content	36.8
% GC content in protein-encoding regions	37.3
% GC content in introns	37.8
% GC content in IGS	31.6
% GC content in rRNA	55.2
% GC content in tRNA	53.1

A total of 23,199 codons represent the 79 different protein-coding genes of the *M. acuminata* chloroplast genome. Among these, 2,350 (10.6%) code for leucine and 269 (1.2%) for cysteine, which are the most frequent and the least frequent amino acids, respectively (Table 2). The 30 different tRNA found in the chloroplast genome correspond to 28 different codons, at least one for each amino acid. Only 7 of the 28 different anticodon tRNAs encoded in the *Musa* plastid genome correspond to the most common codon (where synonymous codons exist). The codon usage is biased towards a high representation of A and T at the third position, as observed in most land plant chloroplast genomes [33].

**Table 2:** Codon usage and codon-anticodon recognition pattern of the *Musa acuminata* chloroplast genome.

Amino acid	Codon	Number	RSCU^a^	Frequency^b^	Amino acid	Codon	Number	RSCU^a^	Frequency^b^
F	TTT	**839**	1.28	**64.19**	A	**GCA**	353	1.13	28.35
F	**TTC**	468	0.72	35.81	A	GCG	122	0.39	9.80
L	**TTA**	**750**	1.91	**31.91**	Y	TAT	**682**	1.58	**78.75**
L	**TTG**	497	1.27	21.15	Y	**TAC**	184	0.42	21.25
L	CTT	465	1.19	19.79	H	CAT	**432**	1.55	**77.56**
L	CTC	158	0.40	6.72	H	**CAC**	125	0.45	22.44
L	**CTA**	324	0.83	13.79	Q	**CAA**	**640**	1.54	**77.11**
L	CTG	156	0.40	6.64	Q	CAG	190	0.46	22.89
I	ATT	**980**	1.47	**48.98**	N	AAT	**850**	1.55	**77.27**
I	**ATC**	384	0.58	19.19	N	**AAC**	250	0.45	22.73
I	ATA	637	0.96	31.83	K	**AAA**	**907**	1.51	**75.52**
M	**ATG**	546	1.00	100.00	K	AAG	294	0.49	24.48
V	GTT	468	1.42	35.51	D	GAT	**763**	1.61	**80.32**
V	**GTC**	168	0.51	12.75	D	**GAC**	187	0.39	19.68
V	**GTA**	**505**	1.53	**38.32**	E	**GAA**	**976**	1.50	**74.90**
V	GTG	177	0.54	13.43	E	GAG	327	0.50	25.10
S	TCT	**512**	1.72	**28.72**	C	TGT	**200**	1.49	**74.35**
S	**TCC**	290	0.98	16.26	C	**TGC**	69	0.51	25.65
S	**TCA**	342	1.15	19.18	W	**TGG**	394	1.00	100.00
S	TCG	161	0.54	9.03	R	**CGT**	326	1.39	23.24
S	AGT	390	1.31	21.87	R	CGC	76	0.33	5.42
S	**AGC**	88	0.30	4.94	R	CGA	312	1.33	22.24
P	CCT	**374**	1.57	**39.33**	R	CGG	105	0.45	7.48
P	CCC	196	0.82	20.61	R	**AGA**	**441**	1.89	**31.43**
P	**CCA**	280	1.18	29.44	R	AGG	143	0.61	10.19
P	CCG	101	0.42	10.62	G	GGT	539	1.38	34.46
T	ACT	**454**	1.54	**38.41**	G	**GGC**	154	0.39	9.85
T	**ACC**	235	0.80	19.88	G	**GGA**	**643**	1.64	**41.11**
T	**ACA**	366	1.24	30.96	G	GGG	228	0.58	14.58
T	ACG	127	0.43	10.74	*	TAA	**41**	1.56	**51.90**
A	GCT	**575**	1.85	**46.18**	*	TAG	20	0.76	25.32
A	GCC	195	0.63	15.66	*	TGA	18	0.68	22.78

a : relative synonymous codon usage.

b : codon frequency relative to each amino acid.

Codons shown in bold complement the anticodons of the tRNAs encoded in the chloroplast genome. Frequencies shown in bold indicate the most common codon (where synonymous codons exist for that amino acid or termination).

The *M. acuminata* chloroplast genome has 18 different intron-containing genes, six of which are tRNA. Most have a single intron except two genes, *clpP* and *ycf3*, which contain two introns. The gene *rps12* is trans-spliced and has the 5′exon in the LSC and two exons in the IR. The *ycf15* and *ycf68* genes were found to have 5 and 7 internal stop codons respectively. This suggests that *ycf15* and *ycf68* have become pseudogenes in *M. acuminata* chloroplast genome. These two pseudogenes were mentioned in very few chloroplast studies and thus were not used in our phylogenetic study. The incomplete duplication of the 5′ end of *ndhA* at the IRa and SSC boundary resulted in two ndhA gene copies: a pseudogene at the boundary of IRb and SSC and a complete copy at the IRa and SSC boundary.

### LSC Orientation Relative to SSC

**Figure 2:**
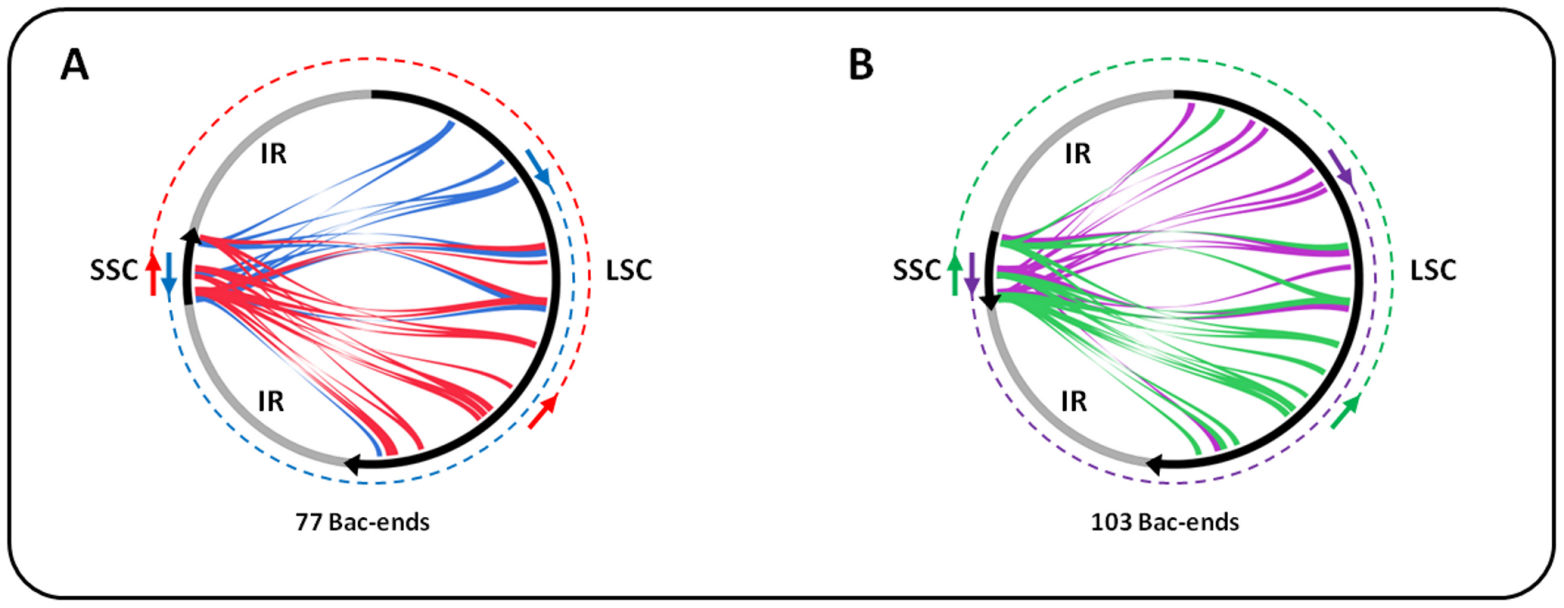
BAC-end-sequences (BESs) mapped on the LSC and SSC *Musa* chloroplast genome. **A**, BESs mapping with the Forward/Reverse (FR), and Reverse/Forward (RF) orientations respectively in blue and red, supporting the SSC orientation relative to the LSC as displayed in the assembled *Musa* chloroplast sequence. **B**, BESs mapping with the Forward/Forward (FF), and Reverse/Reverse (RR) orientations respectively in purple and green, supporting the presence of another form relative to the orientation of the SSC vs LSC in *M. acuminata*.

Due to the inverted repeated regions it was not possible to conclude on the orientation of the SSC relative to the LSC using the 454 and Sanger reads 10 kb paired reads. BAC-end-sequences (BES) were used to orient the SSC relative to LSC. A total of 77 BES, 29 and 48 in the Forward/Reverse and Reverse/Forward orientation respectively, support the orientation presented in this paper (Figure 2A). Another set of 103 BES, 29 and 74 in the Forward/Forward and Reverse/Reverse support a SSC in the reverse complement order (Figure 2B). These results imply that the two forms co-exist in the *M. acuminata* chloroplast genome. This coexistence of two orientation-forms has previously been reported in *Phaseolus vulgaris* [34] and *Zea mays* [35] using RFLP analysis.

### DNA Transfer to the Nucleus

A total of 563 hits (Table 3) of more than 100 bp were found on the eleven chromosomes of *M. acuminata* for a cumulative length of 134,491 bp of the *Musa* nuclear genome (0.41 ‰). This value is situated between those of *Arabidopsis thaliana* (0.17‰) [36] and tomato (0.75‰) [25]. A much higher value (1.85‰) had been found in the rice genome [37]. Matsuo et al. [38], using the rice nuclear genome, reported that the plant nuclear genome is in equilibrium between integration and elimination of the chloroplast genome. The various proportions of inserted chloroplast genome observed in plant species reveal different levels of equilibrium. These variations may result from distinct speed of cp DNA transfer flow to the nucleus or a distinct speed of elimination of the inserted cp DNA or a combination of these two processes.

**Table 3:** Chloroplast genome insertion into the nuclear genome in *Musa acuminata*.

Chromosomes	Nb_hits	Nb_bases		Proportion (%) of all cp insertions	Proportion (%) of the chromosome relative to the total nuclear DNA	
chr01	57	10,468	5.49			8.32
chr02	24	4,637	2.43			6.65
chr03	44	9,629	5.05			9.19
chr04	63	17,752	9.31			9.06
chr05	39	6,993	3.67			8.86
chr06	83	21,129	11.08			10.53
chr07	42	10,838	5.68			8.63
chr08	44	10,482	5.50			10.69
chr09	48	11,125	5.83			10.30
chr10	60	13,659	7.16			10.16
chr11	59	17,779	9.32			7.70

Based on a *per base* insertion value calculated for each plastid base, we showed that the cp DNA inserted in the *Musa* nuclear DNA originate from every part of the chloroplast genome and covers 57.4% of the chloroplast (without IRa) (Figure 1). The highest per base insertion values appeared around the regions carrying the *rpoA* gene and to a further extent in a region containing the *ycf1* gene. In the tomato genome, the *per base* insertion value was also higher in two regions carrying *ycf* genes [25].

Unlike tomato and rice that countain numerous large insertions, only 6 hits of more than 1 kb but not exceeding 2 kb were found on the *M. acuminata* nuclear genome. Chloroplast insertions were found on all chromosomes (Table 3) with a relatively homogeneous distribution unlike the uneven distribution observed in rice [37]. Chromosome 2, with 2.43% of all chloroplast genome insertions, was the chromosome with the least plastid insertion while chromosome 6 was the one having the most abundant plastid insertion (11.08%). The cp DNA insertions into the nuclear genome of *Musa* were evenly distributed over the chromosomes with a reduced number of insertions in pericentromeric regions (Figure S2). However, this may be due to lower assembly quality of this type of regions.

### Phylogenetic Analysis

**Figure 3:**
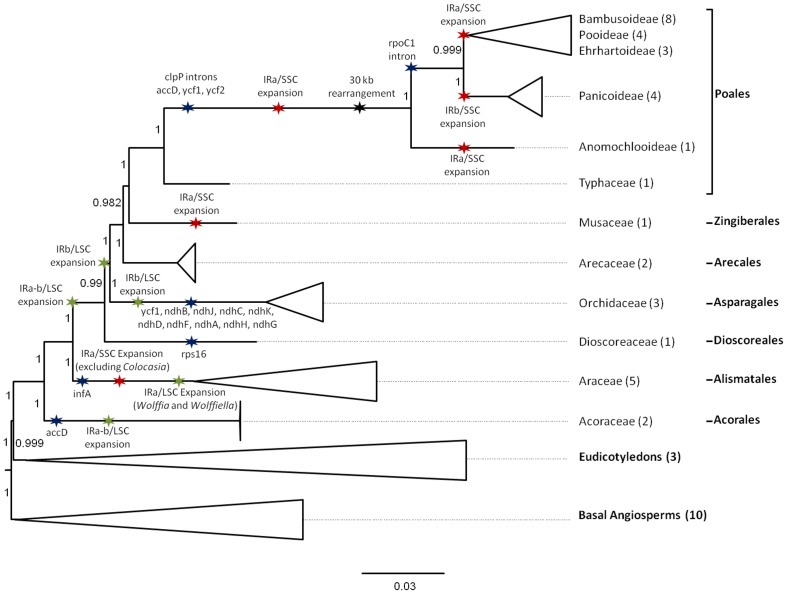
Condensed tree based on the maximum likelihood phylogenetic analysis constructed on 79 chloroplast protein coding genes of 10 basal angiosperms, 35 monocotyledons and 3 dicotyledons. The tree has a -lnL of −527912.066159. Support values for ML are provided at the nodes. Gene losses in all members of the different clades are indicated with blue stars. Putative events of IR expansions/contractions in the monocots are indicated with red and green stars for IR/SSC and IR/LSC boundaries respectively. Major structural rearrangements are indicated with black stars. Numbers indicate aLRT branches support.

A ML phylogenetic analysis was conducted based on 79 protein coding gene from 48 plant taxa. The resulting topology is presented in Figure 3 and Figure S1. All except two nodes are well supported with aLRT statistics higher than 0.98. The first not well supported node with a value of 0.972 is in the Basal Angiosperms group and is positioning *Chloranthus spicatus* at a basal position to the group constituted of *Drimys granadensis*, *Piper cenocladum*, *Calycanthus floridus*, *Magnolia kwangsiensis* and *Liriodendron tulipifera*. The second ambiguous node, with an aLRT value of 0.396, is the relative position of the Bamboo species *Ferrocalamus rimosivaginus* and *Acidosasa purpurea* at the basal position of the others Arundinarieae included in the analysis. Speciation of Zingiberales, Arecales and Poales has long been difficult to resolve and conflicting results have been reported [9]. Our *M. acuminata* chloroplast data positions Zingiberales as sister to the Poales. The Arecales is positioned as sister group to the Poales and Zingiberales in agreement with previous study based on chloroplast genes [6], [8], [9]. However, these results differ from the phylogenetic trees obtained with 93 nuclear single genes, that regroup Zingiberales and Arecales in a sister group to the Poales [11]. Similar incongruence between analyses of single-copy nuclear genes and the chloroplast genes has been observed in the phylogenetic placement of the Malpighiales within the Rosids [39]. These incongruences may be caused by incomplete lineage sorting [40], long-branch attraction phenomenon [41], [42] or chloroplast introgressions between Musaceae and Poales ancestors (see [43] for example). Additional taxa sampling and coalescence-based analyses will be required to resolve this conflict.

### Structural Comparison within Angiosperms

The *M. acuminata* chloroplast genome structure was compared to other angiosperms. Major chloroplast genome structural events (gene losses, IR expansion/contraction and structural rearrangements) and inferred scenarios impacting several Monocotyledons clades are reported on the phylogenetic analysis in Figure 3 (for details on each species and basal angiosperms see Figure S1).

#### Gene content

The *infA* gene has been lost through multiple independent events from at least 24 Angiosperm lineage chloroplast genomes [5]. It is present in the *Musa acuminata* chloroplast genome as well as other Monocotyledons studied so far to the exception of the Alismatale lineage (Figure 3 and [44]). The *accD*, *ycf1* and *ycf2* genes are annotated as functional genes in the *Musa* chloroplastic genome while they have been lost in Poaceae cp genomes [6]. In addition *clpP* and *rpoC1* introns, found in the *Musa* chloroplast genome, have been lost in Poaceae with the exception of the basal Poales *Anomochloa* [45]. The *AccD* and *ycf1* genes have also been lost in the Acoraceae and Orchidaceae. The *ndhB*, *ndhJ*, *ndhC*, *ndhK*, *ndhD*, *ndhF*, *ndhA*, *ndhH*, *ndhG* genes lost in all Orchidaceae genomes sequenced [46]–[48] are all annotated functional in the *Musa* chloroplastic genome as for the *rps16* gene lost in *Dioscorea elephantipes* [49].

#### Structural rearrangements

Dot plot analysis showed that *Musa* chloroplastic genome organization is similar to those found within most angiosperms. The *M. acuminata* chloroplast genome does not present the major structural rearrangement of 30 kb found in Poaceae [6], [50]–[53]. Relative to the *M.* chloroplast genome, this rearrangement consisted in two inversions of 25 and 1 kb respectively and a translocation of about 5 kb all located in the same region.

#### IR expansion/contraction

The most derived chloroplast genome sequenced of Araceae, Bambusoideae, Poideae, Ehrhartoideae, Panicoideae and Anomochlooideae show events of IR/SSC expansion relative to *Amborella trichopoda*. IRs of the *Musa acuminata* chloroplast genome show an extreme extension that includes two additional genes (*rps15* and *ndhH*) plus the full sequence of *ycf1* and 1030 bp of the *ndhA* gene relative to the IR structure of *Amborella trichopoda*. The expansion is made at the IRa/SSC junction and is the largest observed in monocots. In all other monocot groups where IR/SSC expansion is observed, except for the Panicoideae group, the expansion has occurred only at the IRa/SSC junction. The result of these expansions is the inclusion of the whole sequences of *ycf1* and *rps15* in the IRs and a part of the *ndhH* gene except for the Araceae group where *ndhH* is not always included. In the Panicoideae, the IRs contain *rsp15*, and a part of the *ndhF* gene suggesting that the IR/SSC extension has been made in two steps: first an IRa/SSC extension that has included *rps15* in the IR and a second step with an IRb/SSC extension including a part of the *ndhF* gene in the IR. These results suggest that an event of IRa/SSC extension has occurred prior to the divergence of Anomochlooideae, Bambusoideae, Poideae, Ehrhartoideae and Panicoideae including *rps15* gene in the IRs. After the divergence, Anomoclooideae group and Bambusoideae/Poideae/Ehrhartoideae group have been subjected to independent additional IRa/SSC extension to include a part of *ndhH* in the IRs while Panicoideae have been subjected to IRb/SSC extension to include a part of the *ndhF* gene in the IRs. This scenario is similar to the one proposed by Guisinger et al. [6] but it adds the independents events of secondary IRa/SSC expansion in Anomochlooideae and the Bambusoideae/Pooideae/Ehrhatoideae group. This secondary IRa/SSC expansion provides further support for the sister relationship between Bambusoideae, Pooideae and Ehrhatoidea. The phylogenetic position of *M. acuminata* relative to Typhaceae at the basis of Poales and the chloroplastic structure of *Typha* showing no event of IR/SSC expansion suggest that *M. acuminata* has been subjected to an independent event of IRa/SSC expansion relative to Poales. Further investigation should be conducted to determine if this event is common to other Musaceae and the Zingiberales. In the Araceae the most derived species show an IRa/SSC expansion while the basal species *Colocasia esculenta* and the sister group Acoraceae and the Dioscoreaceae, Orchidaceae and Arecaceae do not show IR/SSC expansion. This suggests that this event of IRa/SSC expansion in the Araceae is another independent event. To summarize, three major IRa/SSC expansions may have occurred independently in monocotyledons, one in the Araceae, one in Musaceae and one in the Poaceae. Three secondary independent events of IR/SSC expansion in the Poaceae have occurred, an IRa/SSC expansion in Anomochlooideae, an IRa/SSC expansion in Bambusoideae/Pooideae/Ehrhartoideae group and an IRb/SSC expansion in Panicoideae.

All Monocots sequenced except the most basal Araceae show events of IR/LSC expansion relative to *A. trichopoda* (Figure 3). The Acoraceae and Dioscoreacea display the insertion of the *trnH-GUG* gene at the IRa/LSC boundary and a partial copy of the *rps19* gene at the IRb/LSC boundary. The most derived plastid genomes sequenced of Araceae (*Wolffia australiana* and *Wolffiella lingulata*) only display a partial expansion of the IR including a partial copy of the *rps19* gene at the IRb/LSC boundary. All sequenced plastid genomes belonging to the sister group of the Dioscoreacea (Poales, Zingiberales, Arecales and Asparagales) present the insertion of complete *trnH-GUG* and *rps19* genes located in the LSC of *Amborella* at the IRa/LSC and IRb/LSC boundaries respectively. Asparagales show an additional IRb/LSC expansion as all their whole cp genome sequenced includes a partial copy of the *rpl22* gene. The relative order of *trnH-GUG* and *rps19* genes in IR suggests that in Acoraceae, Dioscoreaceae, Poales, Zingiberales, Arecales and Asparagales the expansion has been made in two steps as proposed by Mardanov et al. [44]: an IRa/LSC expansion leading to the inclusion of the *trnH-GUG* gene in the IR followed with an IRb/LSC expansion leading to the total or partial inclusion of the *rps19* gene in the IR, depending of the group. The structure of the IR/LSC boundary observed in the different clades can be explained by three independent events of IRa-b/LSC expansion, one in Acoraceae, one in the most derived Araceae and one at the basis of the Dioscoreaceae, Poales, Zingiberales, Arecales and Asparagales group. A second round of IRb/LSC expansion has taken place in the last group excluding the Dioscoreaceae leading to the complete inclusion of the *rps19* gene in the IR. A third round of expansion of IRb/LSC expansion has taken place in Asparagales leading to the partial inclusion of *rpl22* gene in the IR as it has been proposed in Wang et al. [54].

### Overview of the Short Tandem Repeats Landscape

Short tandem repeats (also named Simple sequence repeats (SSR)) can exhibit high variation within the same species and are thus considered valuable markers for population genetics [55], [56] and phylogenetic analyses [57]. A total of 112 SSRs were detected in the *Musa* chloroplast genome. Among them, 54 are microsatellites (mono-, di-, tri-, tetra-, penta-, and hexanucleotide repeats) and 58 are minisatellites (unit size ≥10). Minisatellites detected have a unit repeat mean length of 20.8 bp with a minimum of 11 bp and a maximum of 43 bp. The most repeated minisatellite has 14 units of 30 bp repeated tandemly. Among the microsatellites, 39 are exclusively constituted of A/T nucleotides while only one microsatellite is exclusively constituted of C/G nucleotides. Fourteen microsatellites are a mixture of puric and pyrimidic bases.

Sixteen of the homopolymer loci contain multiple A or T nucleotides while only one contains multiple G or C nucleotides. This higher proportion of poly(A)/(T) relative to poly(G)/(C) has also been reported in Poceae [57] and more divergent species such as *Panax ginseng* and *Nicotiana tabacum* [58], *Cucumis sativus* [59], *Magnolia kwangsiensis* [60], *Megaleranthis saniculifolia* [61] or *Sesamum indicum* [62]. However *P. ginseng* and *S. indicum* showed a slightly higher proportion of poly(G)/(C). In *Musa*, among the 10 dinucleotide repeat loci found, 8 are multiple AT or TA and 2 are multiple GA or AG. In Poaceae and *M. saniculifolia*, AT and TA repeats are the most common but others forms are found while only multiple AT or TA are reported in *S. indicum*. In *Musa*, seven trinucleotide repeat loci, fourteen tetranucleotide, five pentanucleotide and one hexanucleotide are found. While in Poaceae tri-, tetra-, penta-, and hexanucleotide repeats are reported [57], no tetra-, penta- and hexanucleotide are reported in the eudicotyledon *S. indicum* and no hexanucleotide are reported in the eudicotyledon *M. saniculifolia*.

### *Musa* Chloroplast PCR Markers

A total of 32 SSR (Table S3) were tested for their polymorphism within a sample of *Musa*. Seven markers appeared monomorphic and 25, 21 and 15 were polymorphic in Musaceae, Eumusa and within the *M. acuminata* sub-species, respectively. The 12 most polymorphic markers were further tested in a sample of six accessions belonging to the chloroplastic group II defined by Carreel et al. [16]. The number of haplotypes detected within our panel is presented in Table 4 for the 12 SSR markers. The average polymorphism level was 4.00, 3.42, 2.92 and 2.33 alleles per marker respectively in Musaceae, Eumusa, *M. acuminata* and within the chloroplastic group II. Among these 12 markers, 9 revealed polymorphism within the chloroplastic group II and showed from 2 to 4 alleles. This new set of chloroplastic PCR markers represents a new, fast and efficient tool for studying the diversity of bananas and the origin of cultivars. Most cultivated bananas are triploids derived from spontaneous hybridization between *M. acuminata* sub-species and a few other *Musa* species but their exact origin is still not completely understood [15], [63]. Their high level of sterility complicates their use in breeding programs. In this context the identification of their fertile progenitors would be very useful for breeders.

**Table 4:** Number of alleles detected within the Musaceae, Eumusa, *M. acuminata* ssp (M. a.) and within the chloroplastic group II samples.

Markers	Musaceae (10)	Eumusa (9)	M. a (8)	cp group II (6)
mMaCIRcp01	4	4	4	3
mMaCIRcp02	2	2	2	2
mMaCIRcp19	4	3	2	1
mMaCIRcp20	5	4	3	3
mMaCIRcp25	4	4	4	4
mMaCIRcp27	4	3	3	2
mMaCIRcp29	4	3	2	1
mMaCIRcp30	5	4	3	2
mMaCIRcp31	3	3	2	1
mMaCIRcp32	4	3	3	3
mMaCIRcp33	5	5	5	4
mMaCIRcp34	4	3	2	2
Average per marker	4.00	3.42	2.92	2.33

The number of accession tested for each group is in parenthesis.

### Conclusion

We assembled, annotated and analyzed the complete chloroplast sequence of banana (*Musa acuminata* ssp *malaccensis*). This first Zingiberale chloroplast (cp) genome was compared to other available monocotyledon cp genomes, providing new insight in their evolution. IR/SSC expansion is particularly pronounced in banana and has occurred independently several times within monocotyledons. The availability of new chloroplast markers within *Musa* opens new perspective to refine the phylogeny of *Musa* and the origin of cultivated triploid bananas.

## Supporting Information

Figure S1 **Maximum likelihood phylogenetic analysis based on 79 chloroplast protein coding genes of 45 basal angiosperms and monocotyledons and 3 Dicotyledons.** The tree has a -lnL of −527912.066159. Support values for ML are provided at the nodes. Gene losses in chloroplast genomes are indicated with red triangles. Green and red stars represent partial or total IR gain of genes belonging respectively to LSC or SSC relative to *A. trichopoda* structure. Green and red minus signs represent loss of one of the two partial or complete gene copies belonging to IR respectively to become member of LSC or SSC relative to *A. trichopoda* structure.
(PDF)

Figure S2 **Localization of cp DNA inserted in the nuclear genome of** ***M. acuminata*** **.**
(PDF)

Table S1 **Chloroplast genomes compared with the** ***M. acuminata*** **chloroplast.**
(PDF)

Table S2 **Distribution of simple sequence repeats (SSRs) loci in the** ***M. acuminata*** **chloroplast genome.**
(PDF)

Table S3 **Makers, associated primer, and expected length tested for the polymorphism analysis.**
(PDF)
