# Supplementary figures and images for: The Complete Chloroplast Genome of Banana (Musa acuminata, Zingiberales): Insight into Plastid Monocotyledon Evolution
Source: PLoS One. 2013 Jun 28;8(6):e67350. doi: 10.1371/journal.pone.0067350 (PMC3696114; doi:10.1371/journal.pone.0067350)

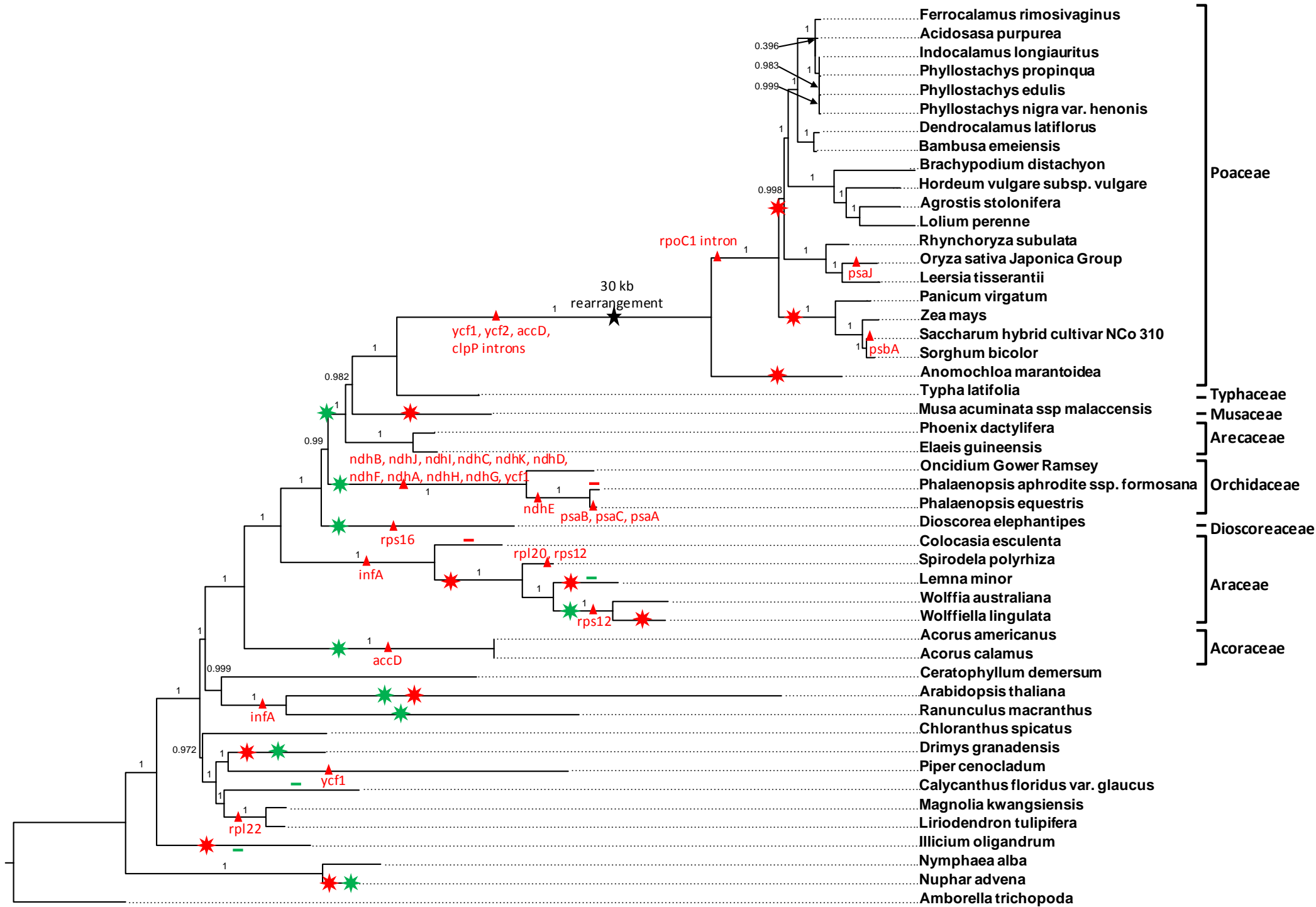

Supplement: Figure S1 — Maximum likelihood phylogenetic analysis based on 79 chloroplast protein coding genes of 45 basal angiosperms and monocotyledons and 3 Dicotyledons. The tree has a -lnL of −527912.066159. Support values for ML are provided at the nodes. Gene losses in chloroplast genomes are indicated with red triangles. Green and red stars represent partial or total IR gain of genes belonging respectively to LSC or SSC relative to A. trichopoda structure. Green and red minus signs represent loss of one of the two partial or complete gene copies belonging to IR respectively to become member of LSC or SSC relative to A. trichopoda structure. (PDF) [file pone.0067350.s001.pdf]

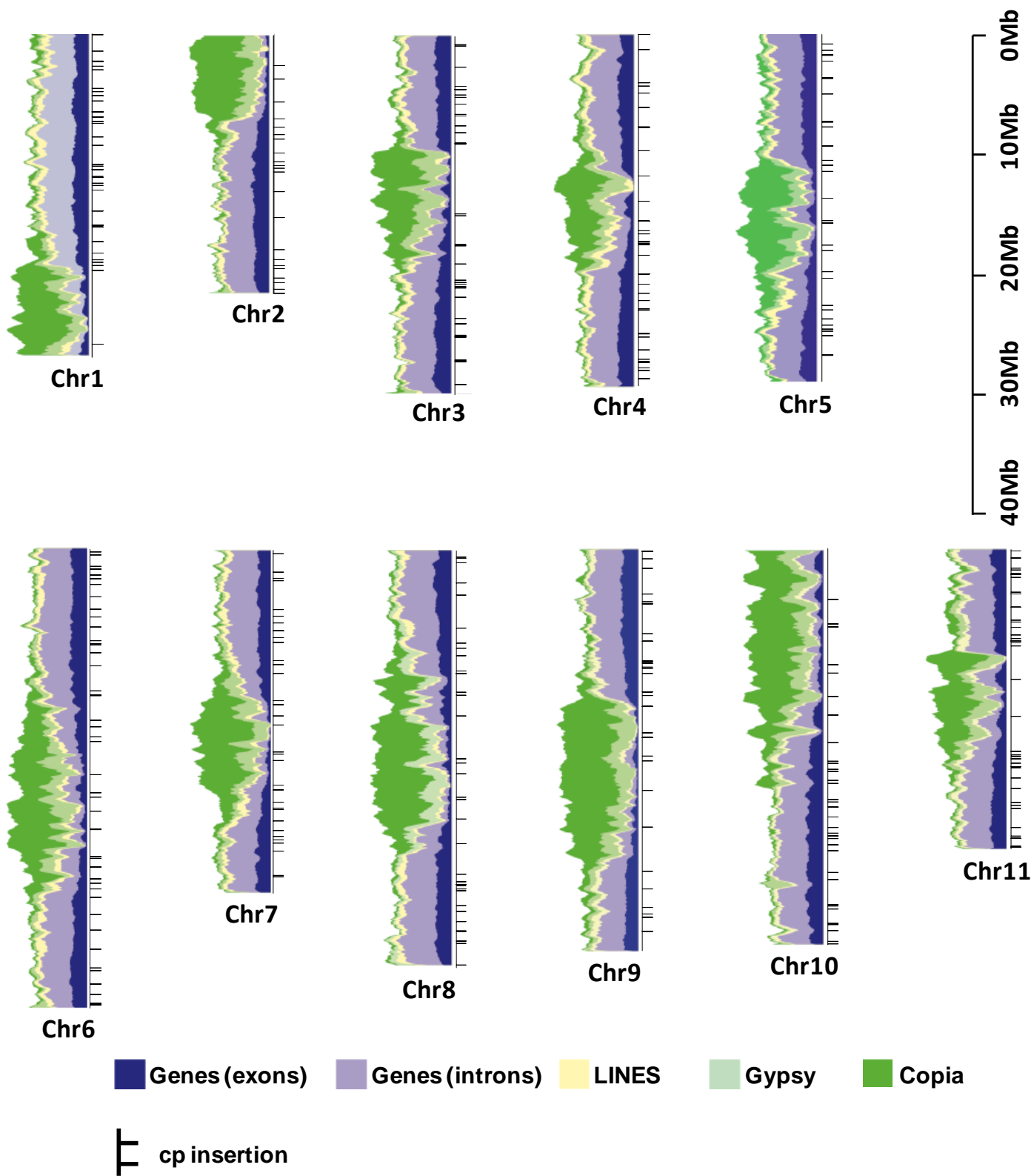

Supplement: Figure S2 — Localization of cp DNA inserted in the nuclear genome of M. acuminata . (PDF) [file pone.0067350.s002.pdf]
