# Supplementary material for: The Complete Chloroplast Genome of Banana (Musa acuminata, Zingiberales): Insight into Plastid Monocotyledon Evolution
Source: PLoS One. 2013 Jun 28;8(6):e67350. doi: 10.1371/journal.pone.0067350 (PMC3696114; doi:10.1371/journal.pone.0067350)
Supplement: Table S1 — Chloroplast genomes compared with the M. acuminata chloroplast. (PDF) [file pone.0067350.s003.pdf]

Taxa	Genome size	GenBank accession
**Monocotyledons**		
Ferrocalamus rimosivaginus	139,467	NC_015831
Acidosasa purpurea	139,697	NC_015820
Indocalamus longiauritus	139,668	NC_015803
Phyllostachys propinqua	139,704	NC_016699
Phyllostachys edulis	139,679	NC_015817
Phyllostachys nigra var. henonis	139,839	NC_015826
Dendrocalamus latiflorus	139,394	NC_013088
Bambusa emeiensis	139,493	NC_015830
Brachypodium distachyon	135,199	NC_011032
Hordeum vulgare ssp. vulgare	136,462	NC_008590
Agrostis stolonifera	136,584	NC_008591
Lolium perenne	135,282	NC_009950
Rhynchoryza subulata	136,303	NC_016718
Oryza sativa Japonica Group	134,525	NC_001320
Leersia tisserantii	136,551	NC_016677
Panicum virgatum	139,619	NC_015990
Zea mays	140,384	NC_001666
Saccharum hybrid cultivar NCo 310	141,182	NC_006084
Sorghum bicolor	140,754	NC_008602
Anomochloa marantoidea	138,412	NC_014062
Typha latifolia	161,572	NC_013823
Phoenix dactylifera	158,462	NC_013991
Elaeis guineensis	156,973	NC_017602
Oncidium Gower Ramsey	146,484	NC_014056
Phalaenopsis aphrodite ssp. Formosana	148,964	NC_007499
Phalaenopsis equestris	148,959	NC_017609
Dioscorea elephantipes	152,609	NC_009601
Colocasia esculenta	162,424	NC_016753
Spirodela polyrhiza	168,788	NC_015891
Lemna minor	165,955	NC_010109
Wolffia australiana	168,704	NC_015899
Wolffiella lingulata	169,337	NC_015894
Acorus americanus	153,819	NC_010093
Acorus calamus	153,821	NC_007407
**Dicotyledons**		
Ceratophyllum demersum	156,252	NC_009962
Arabidopsis thaliana	154,478	NC_000932
Ranunculus macranthus	155,129	NC_008796
**Basal Angiosperms**		
Chloranthus spicatus	157,772	NC_009598
Drimys granadensis	160,604	NC_008456
Piper cenocladum	160,624	NC_008457
Calycanthus floridus var. glaucus	153,337	NC_004993
Magnolia kwangsiensis	159,667	NC_015892
Liriodendron tulipifera	159,886	NC_008326
Illicium oligandrum	148,553	NC_009600
Nymphaea alba	159,930	NC_006050
Nuphar advena	160,866	NC_008788
Amborella trichopoda	162,686	NC_005086
