# Supplementary material for: The Complete Chloroplast Genome of Banana (Musa acuminata, Zingiberales): Insight into Plastid Monocotyledon Evolution
Source: PLoS One. 2013 Jun 28;8(6):e67350. doi: 10.1371/journal.pone.0067350 (PMC3696114; doi:10.1371/journal.pone.0067350)
Supplement: Table S2 — Distribution of simple sequence repeats (SSRs) loci in the M. acuminata chloroplast genome. (PDF) [file pone.0067350.s004.pdf]

Region location	Unit length	SSR	Size	Start	End
LSC	5	(CTTAT)3	15	25	39
LSC	5	(AATAA)3	15	4300	4314
LSC	4	(ATT)4	12	5432	5443
LSC	1	(A)15	15	5455	5469
LSC	4	(ATTG)3	12	5893	5904
LSC	1	(C)10	10	8004	8013
LSC	4	(TATT)5	20	16007	16026
LSC	3	(TAT)4	12	16049	16060
LSC	3	(TAT)4	12	16061	16072
LSC	5	(TATTA)3	15	16073	16087
LSC	3	(TAA)5	15	16584	16598
LSC	1	(T)12	12	20615	20626
LSC	2	(AT)5	10	21986	21995
LSC	4	(AATA)3	12	30171	30182
LSC	2	(TA)6	12	31687	31698
LSC	5	(TTTAT)3	15	33492	33506
LSC	1	(A)10	10	34538	34547
LSC	4	(TTTG)3	12	35573	35584
LSC	1	(T)11	11	40036	40046
LSC	5	(TATAG)3	15	49486	49500
LSC	4	(CAAA)3	12	50026	50037
LSC	2	(AT)9	18	51235	51252
LSC	2	(TA)7	14	51297	51310
LSC	3	(AAT)4	12	54057	54068
LSC	1	(T)11	11	58405	58415
LSC	1	(T)12	12	58766	58777
LSC	1	(A)10	10	60414	60423
LSC	2	(TA)5	10	60489	60498
LSC	4	(ATAA)3	12	63130	63141
LSC	1	(A)10	10	64943	64952
LSC	4	(GAAA)3	12	67045	67056
LSC	4	(TCTT)3	12	68079	68090
LSC	3	(TTC)4	12	71867	71878
LSC	2	(TA)6	12	74114	74125
LSC	4	(AAGA)3	12	74683	74694
LSC	1	(T)12	12	75118	75129
LSC	1	(T)14	14	75548	75561
LSC	4	(AAAT)3	12	76130	76141
LSC	1	(T)13	13	85019	85031
IR	2	(GA)5	10	94674	94683
IR	3	(AGA)10	30	116275	116304
IR	3	(AGA)4	12	116527	116538
IR	6	(AAGCAG)5	30	117051	117080
IR	1	(T)10	10	117236	117245
IR	1	(A)12	12	117383	117394
IR	1	(A)10	10	117875	117884
IR	1	(A)15	15	120011	120025
IR	2	(AG)5	10	121964	121973
SSC	2	(TA)5	10	126873	126882
SSC	2	(AT)5	10	128655	128664
SSC	4	(AATA)3	12	129058	129069
SSC	4	(TTTA)3	12	131797	131808
SSC	1	(A)10	10	132407	132416
SSC	4	(AATA)3	12	134345	134356
