# Supplementary material for: The Complete Chloroplast Genome of Banana (Musa acuminata, Zingiberales): Insight into Plastid Monocotyledon Evolution
Source: PLoS One. 2013 Jun 28;8(6):e67350. doi: 10.1371/journal.pone.0067350 (PMC3696114; doi:10.1371/journal.pone.0067350)
Supplement: Table S3 — Makers, associated primer, and expected length tested for the polymorphism analysis. (PDF) [file pone.0067350.s005.pdf]

Markers	Forward	Reverse	length
mMaCIRcp01*	CCCCCAGAAACGTATAGGAG	TTTCCCTTCGAATTCTGTCA	234
mMaCIRcp02*	TAACCTCCCCAACCCTTCTT	GTGAATCCATGGAGGGTCAT	222
mMaCIRcp03	CCTGCAAGTAAAGGGGGTTC	TTTCCGTGAGTCCTCGAAAT	197
mMaCIRcp05	TGTTTTGTTCTTCCGCCAAT	TTGAACCCGGAATTGTCCTA	200
mMaCIRcp06	TCGAGATAGATCGGGCGTTA	CGGGAGCATAATCTCACTTTG	270
mMaCIRcp07	TCTCACTTTGTCTTGGGTTCTC	TCCCTAGACATTTTACCCCATA	277
mMaCIRcp08	AAAAAGATTGGGCCGATTG	CCAATCCCAAGGATCCATAA	201
mMaCIRcp09	TGAAATGATAATTGCAACGAAA	GGGCACAACTGGTACATTCC	198
mMaCIRcp10	TGGCCAAGGGTAAAGATGTC	TCCGACCAAAATAGGCTTTG	391
mMaCIRcp12	TTATCGGCTGTCTTGCGTTT	TACCGGGGATTTCGTGTAAA	211
mMaCIRcp13	CATCCAGCAGGAATTGAACC	CTCAATTCGACGATCCAGAA	462
mMaCIRcp14	GATTGATTGGTCCGAGGCTA	CCGGTCTTTGGGAAGTATCA	299
mMaCIRcp15	CCTTGTTCATTCGCAACAAA	TCGAATTGGAAAACGGAAAG	182
mMaCIRcp16	GCTTGTGGGGTCAAATCAAT	CCAATCACCGTTCACCTTTT	216
mMaCIRcp17	ACAAGTTCCCGGATGACAAG	GGATCCACTTTTTGGGGAAT	388
mMaCIRcp18	TGGGTTTCTACCAATGAGCA	TTGCTCATTCTCATCGTTGC	165
mMaCIRcp19*	GGACCGTATCGTGGAACAAT	GCGGATTCTTTTCATGTTCA	226
mMaCIRcp20*	CGAAACGGGTGGTGATCTAT	GGGGAATGAACATTTGTTTGA	217
mMaCIRcp21	AACGGAACTCCCTTTTGGTT	TTTGAATGGTCGATTCGTGA	380
mMaCIRcp22	GATTGGATGGGAATGAATCG	CCCTCTTTTTCTTGGTTGGA	467
mMaCIRcp23	TGCACCAGAACAACTGGAAA	CATCATTCGCATACCTGTGG	211
mMaCIRcp24	TGAAAATTCTTTTGTTTCTTATATGC	TTTTCTAACGATTTCGACACCT	168
mMaCIRcp25*	AATAACGGGACCAAAACC	TCCTTCCTTCCATTCTCA	287
mMaCIRcp26	TTTCTGTTTCCGGTGGTA	ATCTTACCCGGATCTTCG	223
mMaCIRcp27*	CGGTTCAGGGTACGAATA	CCCCAAAAGTAAAAAGTGG	207
mMaCIRcp28	AACGTCGATGGAGACGTA	CATTTGATTCGTCGATCC	127
mMaCIRcp29*	AGTTGGTACCACCCAACC	GGCGGAAATCCAATATCT	283
mMaCIRcp30*	AACAAACATTGGGTTTGG	AGTCCCTCCCTACAACTCA	279
mMaCIRcp31*	TCAACGAATGAAGCAGGT	TATATGCGTTTCCGGGTA	292
mMaCIRcp32*	ACCCCCGACACATAAAAT	CCGCTTCTATGGGATCTT	275
mMaCIRcp33*	GGATGCATACGGTTCAAA	AAAGGCCCATTCAGAAAC	262
mMaCIRcp34*	TGGTGCCTCCTAATTTTG	CGGGAATTGAGACAGTTG	250

*Highly polymorphic markers tested for the splitting of chloroplastic group II in *M. acuminata* subspecies.
